# Identification of Plasma hsa_circ_0005397 and Combined With Serum AFP, AFP-L3 as Potential Biomarkers for Hepatocellular Carcinoma

**DOI:** 10.3389/fphar.2021.639963

**Published:** 2021-02-19

**Authors:** Ruoyu Liu, Yi Li, Anqi Wu, Mingzhu Kong, Weijia Ding, Zeyang Hu, Lin Chen, Weihua Cai, Feng Wang

**Affiliations:** ^1^ Department of Laboratory Medicine, Affiliated Hospital of Nantong University, Jiangsu, China; ^2^ Department of Laboratory Medicine, School of Public Health, Nantong University, Jiangsu, China; ^3^ Department of Gastroenterology and Laboratory Medicine, Nantong Third Hospital Affiliated to Nantong University, Jiangsu, China

**Keywords:** hepatocellular carcinoma, circular RNA, hsa_circ_0005397, alpha-fetoprotein, biomarker

## Abstract

**Background:** Mounting evidence has demonstrated that circular RNA (circRNA) plays crucial roles in the occurrence and development of hepatocellular carcinoma (HCC). However, the expression pattern and clinical application value of plasma circRNA in HCC are still largely unknown. Herein, we explored the role of plasma hsa_circ_0005397 in diagnosis and prognosis of HCC.

**Methods:** The expression level of plasma hsa_circ_0005397 was measured by quantitative real-time polymerase chain reaction (qRT-PCR). The identification and origin of plasma hsa_circ_0005397 were confirmed by RNase R assay, Sanger sequencing and HCC cell culture. In addition, its diagnostic value was assessed by receiver operating characteristic (ROC) curve and prognostic value was evaluated by dynamics monitoring and Kaplan–Meier curve analyses in HCC patients.

**Results:** The expression of plasma hsa_circ_0005397 was higher in patients with HCC than that in patients with benign liver diseases and healthy controls (both *p* < 0.05). Moreover, it was closely correlated with tumor size (*p* = 0.020) and TNM stage (*p* = 0.006) of HCC patients. The area under the ROC curve of plasma hsa_circ_0005397 was 0.737 and 95% confidence interval was 0.671–0.795. Furthermore, the combination of plasma hsa_cic_0005397, serum AFP and AFP-L3 could improve the diagnostic sensitivity of HCC. Additionally, dynamic monitoring plasma hsa_cic_0005397 might help us predict recurrence or metastasis in HCC patients after surgical resection. Besides, the increased plasma hsa_cic_0005397 was closely correlated with shorter overall survival of HCC patients (*p* = 0.007).

**Conclusion:** Plasma has_circ_0005397 represents a novel noninvasive biomarker for HCC. Moreover, the combination of plasma hsa_cic_0005397, serum AFP and AFP-L3 might improve the diagnostic value for HCC.

## Introduction

Hepatocellular carcinoma (HCC), with high morbidity and high mortality, remains one of the most common malignant tumors worldwide. In China, Hepatitis B or C viral (HBV or HCV) infection contributes to the important risk factor for HCC and accounts for a great proportion of cancer-related death every year (Mak et al., 2018; Fu et al., 2019). In the past decades, much progress has been made in methods and strategies of diagnosis and treatment of HCC. However, the five-year survival rate of HCC has not essentially improved due to its frequent recurrence and metastasis. To date, serum alpha-fetoprotein (AFP) has been routinely used for clinical detection of HCC, whereas, the diagnostic value remains suboptimal (Sun et al., 2019; Ding et al., 2020). As a result, these challenges make it urgent to find novel biomarkers for early diagnosis, assessment of treatment and prognosis of HCC.

Circular RNA (circRNA), a special class of non-coding RNA molecules, can be widely expressed in various cells and tissues. Unlike linear RNA, circRNA has not 5′-end cap and 3′-end poly (A) tail, but owns a closed-loop structure. Thus, it is more stable and difficult to degrade than linear RNA, due to its less susceptible to exonucleases (Ebbesen et al., 2017; Huang et al., 2019; Yin et al., 2019). Recent studies have found that circRNA plays important role in tumorigenesis and pathogenesis of the majority of malignancies via diverse mechanisms. For example, dysregulated circRNA can act as a microRNA sponge or directly bind to proteins, thereby modulating the expression of downstream target gene, and resulting in the occurrence and development of cancer (Yao et al., 2019; Zhu et al., 2019; Jie et al., 2020; Xiao et al., 2020). Moreover, circRNA is spatial and temporal-specific expression and it also exists in different body fluids, such as blood, saliva and urine, etc. (Lin et al., 2019; Han et al., 2020). Thus, circulating circRNA can be used as a promising biomarker for cancer, including HCC.

To date, a few studies have investigated the role of plasma circRNA in diagnosis of HCC (Zhu et al., 2020; Yu et al., 2020). However, the potential clinical application value of plasma circRNA in HCC is still elusive. We firstly searched from GSE97332 database and GSE97508 database, and found that hsa_circ_0005397 was upregulated in HCC in both two databases. Hsa_circ_0005397was discovered as a circRNA to its host gene RHOT1 from circBank database (http://www.circbank.cn/). Moreover, it has been reported that circRHOT1 as a conserved circRNA is dramatically over-expressed in HCC and promotes cancer progression by initiation of NR2F6 expression (Wang et al., 2019). These findings indicated that hsa_circ_0005397, as one of circRHOT1s, might play important role in HCC. In the current study, we verified that hsa_circ_0005397 was upregulated in the plasma samples of HCC patients. The clinical performance of plasma has_circ_0005397 for HCC was further investigated and the results showed that it might act as a potential noninvasive biomarker for diagnosis and prognosis of HCC.

## Materials and Methods

### Patient Selection and Sample Collection

A total of 208 participants from January 2015 to November 2016 in Nantong Third Hospital Affiliated to Nantong University and Affiliated Hospital of Nantong University were recruited in this study, including 89 patients with HCC, 40 patients with benign liver diseases, and 79 healthy controls. Demographic and clinical characteristics of study population are summarized in Table 1. Patients with HCC were diagnosed based on histopathological examination and had not received any preoperative radiotherapy or chemotherapy. The clinicopathological stage was determined according to the International Anti-Cancer Association's tumor-lymph node-metastasis (TNM) staging system, and the degree of differentiation was evaluated according to the World Health Organization classification system. The EDTA-K2 anticoagulant plasma from all participants were collected in EP tubes without RNase and DNase, and frozen in −80°C refrigerator for further use.

**TABLE 1:** Demographic and clinical characterization of study population.

Variables	HCC (*n* = 89)	Benign (*n* = 40)	Healthy (*n* = 79)
Age (years)			
Median (range)	58 (40–79)	54 (36–74)	51 (32–70)
Gender			
Male	78	31	64
Female	11	9	15
HBV infection			
Positive	59	28	0
Negative	30	12	79
Hepatocirrhosis			
Presence	47	20	0
Absence	42	20	79
Serum AFP (ng/ml)			
≤200	27	36	79
>200	62	4	0
Tumor size (cm)			
≤5	44		
>5	45		
Tumor differentiation			
Well + Moderate	51		
Poor	38		
TNM stage			
I + II	40		
III + IV	49		

### Cell Culture

Cell lines were purchased from the Type Culture Collection Cell Committee of the Chinese Academy of Sciences (Shanghai, China), including 4 human HCC cell lines (SK-Hep1, SMMC-7721, BEL-7404, Huh7) and a normal liver cell line LO2. Cells were cultured in DMEM medium (Corning, USA) containing 10% fetal bovine serum (FBS) and 1% penicillin-streptomycin, maintained in a humidified incubator with 5% CO_2_ at 37°C.

### RNA Extraction, Reverse Transcription (RT) and Real-Time Quantitative Polymerase Chain Reaction (qRT-PCR)

Total plasma RNAs were extracted using the Trizol LS (Ambion, United States) in accordance with the manufacturer’s instructions. RNA concentration was detected with NanoDrop 2000 ultra-micro spectrophotometer (Thermo Fisher Scientific, United States). Reverse transcription of plasma total RNA was performed by the Revert Aid First Strand cDNA Synthesis Kit (Thermo Fisher Scientific, United States). Plasma circRNAs were measured with Plus SYBR real-time PCR mixture (Bio Teke, Beijing, China) by qRT-PCR analyses. All reactions were performed using Roche LightCycler 480 (Roche, Switzerland) according to the following protocol: 95°C for 15 s, then 45 cycles of 60°C for 30 s and 72°C for 30 s. The internal reference was 18S rRNA. The sequence of primers are as follows: hsa_circ_0005397, 5′-GACAAAGACAGCA GGTTCCT-3′ (forward) and 5′-CTC​TGT​TCT​GCT​TCT​GAG​TA-3′ (reverse); hsa_ circ_0006302, 5′-GCC​TAC​ATG​ATC​GAG​GAT​A-3′ (forward) and 5′-GGATCTG GGTGTTCCTTTAC-3′ (reverse); hsa_circ_0088494, 5′-GCCCACTCCCTAGCAA CTGA-3′ (forward) and 5′-CCA​ACT​CCA​GCA​CAA​TGT​TC-3′ (reverse); hsa_circ_ 0083766, 5′-AGA​ACC​TGA​GTC​GGA​CTT​TCA-3′ (forward) and 5′- GGGGACATG TTGGGATTTGC-3′ (reverse); 18S rRNA, 5′-GTA​ACC​CGT​TGA​ACC​CCA​TT-3′ (forward) and 5′-CCATCCAATCGGTA GTAGCG-3′ (reverse). The relative expression levels of plasma circRNAs were calculated using the 2^−ΔΔCT^ method.

### Identification of circRNA

Plasma total RNA was treated with or without RNase R (Epicenter, United States), and purified with RNeasy Min Elute Cleanup Kit (QIAGEN, Germany). Reverse transcription was conducted with random 6-mers or oligo (dT) primers. After qRT-PCR, the products were subjected to 2% agarose gel electrophoresis and sent for Sanger sequencing (Sangon, Shanghai, China).

### Detection of Serum AFP and AFP-L3

Serum AFP-L3 levels were measured using the AFP-L3 detection kit (Rejing, Beijing, China) and serum AFP concentration was determined by chemiluminescence immunoassay using ARCHITECT i2000SR analyzer (Abbott, United States).

### Statistical Analysis

Statistical analyses were performed using SPSS 20.0 and graphs were drawn using GraphPad Prism 7.0 software (GraphPad Software Inc., California, United States). Differences in plasma circRNA concentrations were estimated by independent samples *t*-test, one-way analysis of variance (ANOVA) and Fisher’s exact test, as appropriate. Receiver operator characteristic (ROC) curve was drawn to evaluate the diagnostic value of plasma hsa_circ_0005397 for HCC. In addition, Kaplan–Meier survival curve was constructed to evaluate survival data. A two-sided *p* < 0.05 was considered as statistically significant.

## Results

### Screening of Candidate Plasma circRNA in Patients With HCC

**FIGURE 1:**
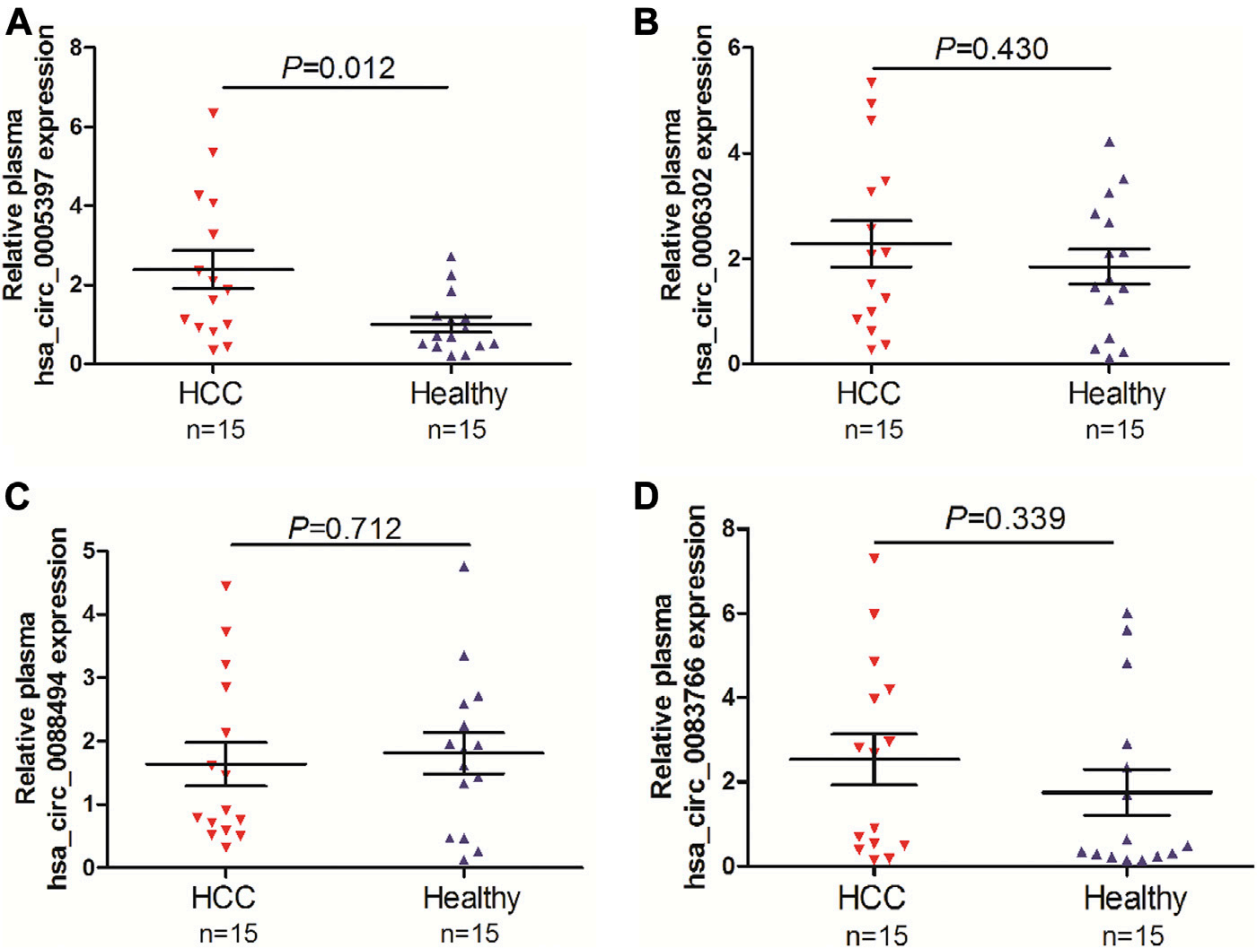
Screening of candidate plasma circRNA in patients with HCC. Detection of plasma expression levels of 4 candidate circRNAs, hsa_circ_0005397 **(A)**, hsa_circ_0006302 **(B)**, hsa_circ_0088494 **(C)** and hsa_circ_0083766 **(D)**, by qRT-PCR analyses in 15 HCC vs. 15 healthy controls.

To screen the candidate plasma circRNA in patients with HCC, we investigated 4 dysregulated circRNAs from GSE97332 database and GSE97508 database, hsa_circ_0005397, hsa_circ_0006302, hsa_circ_0088494 and hsa_circ_0083766, by qRT-PCR analyses in the plasma of 15 patients with HCC and 15 healthy volunlteers. The results showed that plasma hsa_circ_0005397 was over-expressed in patients with HCC (*p* = 0.012, Figure 1A). However, there was no significant difference of plasma hsa_circ_0006302, hsa_circ_0088494 and hsa_circ_0083766 expressions between HCC group and healthy group (all *p* > 0.05, Figures 1B–D). Therefore, we further investigated the clinical significance of plasma hsa_circ_0005397 in HCC patients.

### Verification of Increased Plasma hsa_circ_0005397 in Patients With HCC

**FIGURE 2:**
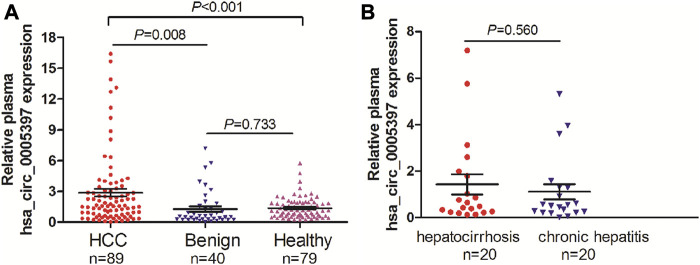
Plasma hsa_circ_0005397 is increased in HCC patients. **(A)** Detection of plasma hsa_circ_0005397 expression levels in 89 patients with HCC, 40 patients with benign liver diseases and 79 healthy controls. **(B)** Detection of plasma hsa_circ_0005397 expression levels in 20 patients with chronic hepatitis and 20 patients with hepatocirrhosis.

Based on the above-mentioned investigation, we then measured plasma hsa_circ_0005397 levels in 89 patients with HCC, 40 patients with benign liver diseases and 79 healthy controls by using qRT-PCR analyses. The results showed that the plasma hsa_cic_0005397 levels in HCC patients were obviously higher than that in patients with benign liver lesions and healthy controls, *p* = 0.008 and *p* < 0.001, respectively. Whereas, there was no statistically significant difference of plasma hsa_cic_0005397 level between the benign liver disease group and the healthy control group, *p* = 0.733 (Figure 2A). Besides, 40 patients with benign liver diseases were comprised of 20 patients with chronic hepatitis and 20 patients with hepatocirrhosis. We also found plasma hsa_cic_0005397 level had no obvious change between the chronic hepatitis group and the hepatocirrhosis group, *p* = 0.560 (Figure 2B).

### The Identification of Plasma hsa_circ_0005397

**FIGURE 3:**
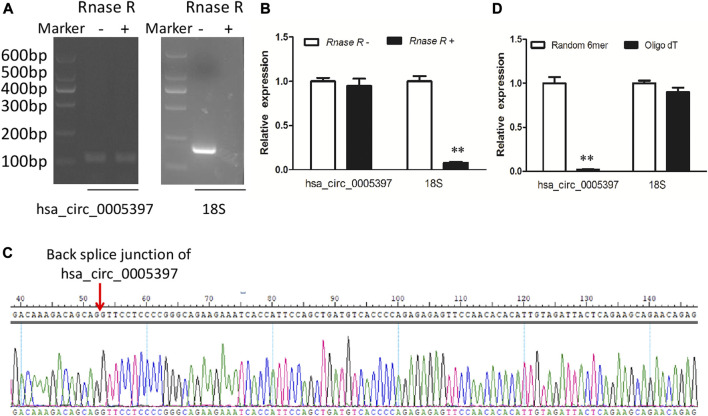
The identification of plasma hsa_circ_0005397. **(A)** Total RNA was treated or not treated with RNase R, gel electrophoresis of the RT-PCR products was performed. **(B)** After pretreated or not pretreated with RNase R, the expression levels of plasma hsa_circ_0005397 and 18S rRNA were detected by qRT-PCR. **(C)** The qRT-PCR product of the plasma hsa_circ_0005397 was verified by Sanger sequencing. **(D)** The cDNA of hsa_circ_0005397 and 18S rRNA were transcribed with random 6-mer or oligo dT primers and then measured by qRT-PCR, **p < 0.01.

Plasma hsa_circ_0005397 was further investigated by RNase R digestion and qRT-PCR. Total RNA was isolated from plasma and treated with RNase R. The hsa_circ_0005397 was amplified by our designed primers and the product was resistant to RNase R digestion. The 18S rRNA, as a linear RNA, was used as a negative control (Figures 3A,B). The PCR product was then verified by Sanger sequencing, which included back splice junction site of hsa_circ_0005379 (Figure 3C), indicating that the primers was specific divergent primers targeting hsa_circ_0005397. Because circRNA lacks a poly A tail, we reverse-transcribed plasma RNA with random 6-mers or oligo (dT) primers and then performed qRT-PCR assays. The results showed that hsa_circ_0005397 could effectively be transcribed by random 6-mer but not oligo (dT) primers, using the 18S rRNA as a positive control (Figure 3D), revealing that hsa_circ_0005397 had a complete closed loop structure but no poly A tail. Taken together, these results suggested that hsa_circ_0005397 was stable in the plasma of HCC patients.

### The Origin of Circulating hsa_circ_0005397

**FIGURE 4:**
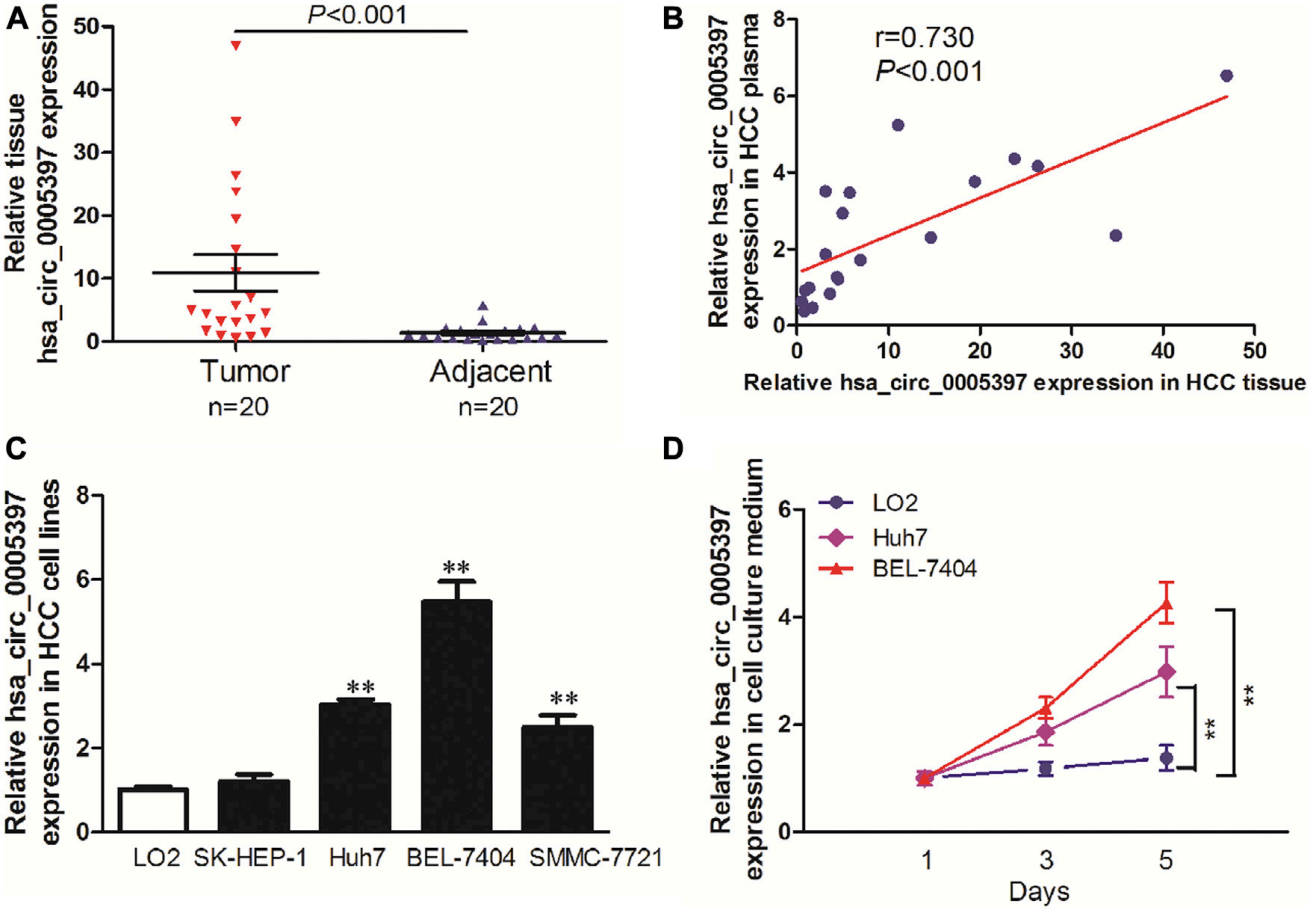
The origin of circulating hsa_circ_0005397. **(A)** The expression levels of hsa_circ_0005397 in HCC tissues and corresponding adjacent normal tissues. **(B)** The correlation between plasma hsa_circ_0005397 expression levels and tissue hsa_circ_0005397 expression levels. **(C)** The expression levels of hsa_circ_0005397 in 4 HCC cell lines. **(D)** Hsa_circ_0005397 was released from tumor cells into the cell culture medium in a time-dependent manner, **p < 0.01.

To explore the origin of circulating hsa_circ_0005397, we firstly examined hsa_circ_0005397 expression level in paired tumor tissues and their adjacent normal tissues of 20 HCC patients from training cohort. The results showed that the expression level of hsa_circ_0005397 was significantly higher in HCC tissues than that in non-tumor tissues, *p* < 0.001 (Figure 4A). Moreover, we also found a positive correlation between plasma hsa_circ_0005397 expression level and tissue hsa_circ_0005397 expression level in 20 HCC patients, *r* = 0.730, *p* < 0.001 (Figure 4B). In addition, we investigated the expression level of hsa_circ_0005397 in four human HCC cell lines (SK-Hep1, Huh7, BEL-7704 and SMMC7721) and a normal liver cell line (LO2). As shown in Figure 4C, hsa_circ_0005397 was frequently upregulated in HCC cell lines, especially in BEL-7704 and Huh7 cell lines. Meanwhile, we conjectured whether hsa_circ_0005397 could be secreted by HCC cells. The expression levels of hsa_circ_0005397 in BEL-7704 and Huh7 cell culture medium and LO2 cell culture medium were analyzed. As expected, the expression levels of hsa_circ_0005397 in the culture medium of BEL-7704 and Huh7 cell lines were conspicuously increased in a time-dependent manner while no significant change was observed in the cell culture medium of LO2 (Figure 4D). These results indicated that plasma hsa_circ_0005397 might be released from tumor cells into the blood circulatory system.

### Association of Plasma hsa_circ_0005397 with Clinicopathological Characteristics in HCC Patients

To determine whether plasma hsa_cic_0005397 level was related to the clinicopathological characteristics of HCC, HCC cases were stratified by age, gender, hepatitis B virus (HBV) infection, hepatocirrhosis, serum AFP, tumor size, differentiation degree, and TNM stage. According to the median level = 1.87 of plasma hsa_cic_0005397, 89 HCC patients were subdivided into two groups (high group, *n* = 44 and low group, *n* = 45). The Fisher's exact test statistical analysis revealed that the expression level of plasma hsa_cic_0005397 was positively correlated with tumor size (*p* = 0.020) and TNM stage (*p* = 0.006). However, there was no significant correlation of plasma hsa_cic_0005397 with other clinicopathological features, such as age, gender, HBV infection, hepatocirrhosis, serum AFP and tumor differentiation, all *p* > 0.05 (Table 2).

**TABLE 2:** Association of plasma hsa_circ_0005397 with clinicopathologic characteristics of HCC patients.

Variables	*n*	hsa_circ_0005397		
		High (*n* = 44)	Low (*n* = 45)	*p* value
Age (year)				0.399
≤58	45	20	25	
>58	44	24	20	
Gender				0.522
Male	78	40	38	
Female	11	4	7	
HBV infection				0.117
Positive	59	33	26	
Negative	30	11	19	
Hepatocirrhosis				0.291
Present	47	26	21	
Absent	42	18	24	
Serum AFP (ng/ml)				0.167
≤200	27	10	17	
>200	62	34	28	
Tumor size (cm)				0.020*
≤5	44	16	28	
>5	45	28	17	
Tumor differentiation				0.088
Well + Moderate	51	21	30	
Poor	38	23	15	
TNM stage				0.006*
I–II	40	13	27	
III–IV	49	31	18	

Fisher's exact test, *p < 0.05.

### Diagnosis Utility of Plasma hsa_circ_0005397 for HCC

**FIGURE 5:**
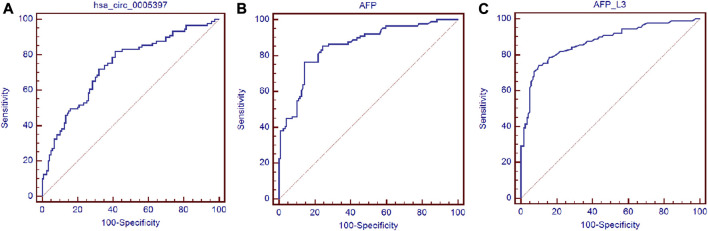
Diagnosis utility of plasma hsa_circ_0005397 for HCC. **(A**–**C)** Construction of ROC curve to compare the diagnostic performance of plasma hsa_circ_0005397, serum AFP and AFP-L3 to discriminate HCC from benign liver diseases and healthy controls.

Next, ROC curve was drawn to assess the diagnostic performance of the plasma hsa_cic_0005397 in differentiating HCC from benign liver diseases and healthy controls. As shown in Figure 5A, the area under the curve (AUC) (95% confidence interval (CI)) of plasma hsa_cic_0005397 was 0.737 (0.671–0.795), *p* < 0.001. When the cut-off value was defined at 0.914, sensitivity was 82.0%, specificity was 58.8%, and the Youden index was 0.408. As serum AFP and AFP-L3 are widely used in clinical practice for screening and auxiliary diagnosing of HCC, we also compared their diagnostic values with plasma hsa_cic_0005397. The results showed that the AUCs of serum AFP and AFP-L3 were 0.855 (95% CI, 0.800–0.900, *p* < 0.001) and 0.872 (95% CI, 0.819–0.915, *p* < 0.001), respectively, both of them were slightly better than plasma hsa_cic_0005397 (Figures 5B,C). We then investigated the diagnostic performance by combining with plasma hsa_cic_0005397, serum AFP and AFP-L3. As compared with hsa_cic_0005397, AFP or AFP-L3 alone, the diagnostic sensitivity was improved by the combination of hsa_cic_0005397 + AFP (89.9%) and hsa_cic_0005397+AFP-L3 (87.6%), especially by a combination of these three biomarkers (93.3%, Table 3).

**TABLE 3:** Diagnostic value of HCC by plasma hsa_circ_0005397, serum AFP and AFP-L3.

Biomarker	Sensitivity (%)	Specificity (%)	Positive predictive value (%)	Negative predictive value (%)	Efficiency (%)
hsa_circ_0005397	82.0 (73/89)	58.8 (70/119)	59.8 (73/122)	81.4 (70/86)	68.8 (143/208)
AFP	76.4 (68/89)	85.7 (102/119)	80.0 (68/85)	82.9 (102/123)	81.7 (170/208)
AFP-L3	74.2 (66/89)	89.9 (107/119)	84.6 (66/78)	82.3 (107/130)	83.2 (173/208)
hsa_circ_0005397 + AFP	89.9 (80/89)	57.1 (68/119)	61.1 (80/131)	88.3 (68/77)	71.2 (148/208)
hsa_circ_0005397 + AFP-L3	87.6 (78/89)	58.0 (69/119)	60.9 (78/128)	86.3 (69/80)	70.7 (147/208)
hsa_circ_0005397 + AFP + AFP-L3	93.3 (83/89)	55.5 (66/119)	61.0 (83/136)	91.7 (66/72)	71.6 (149/208)

### Dynamic Analysis of Plasma hsa_circ_0005397 in Patients With HCC

**FIGURE 6:**
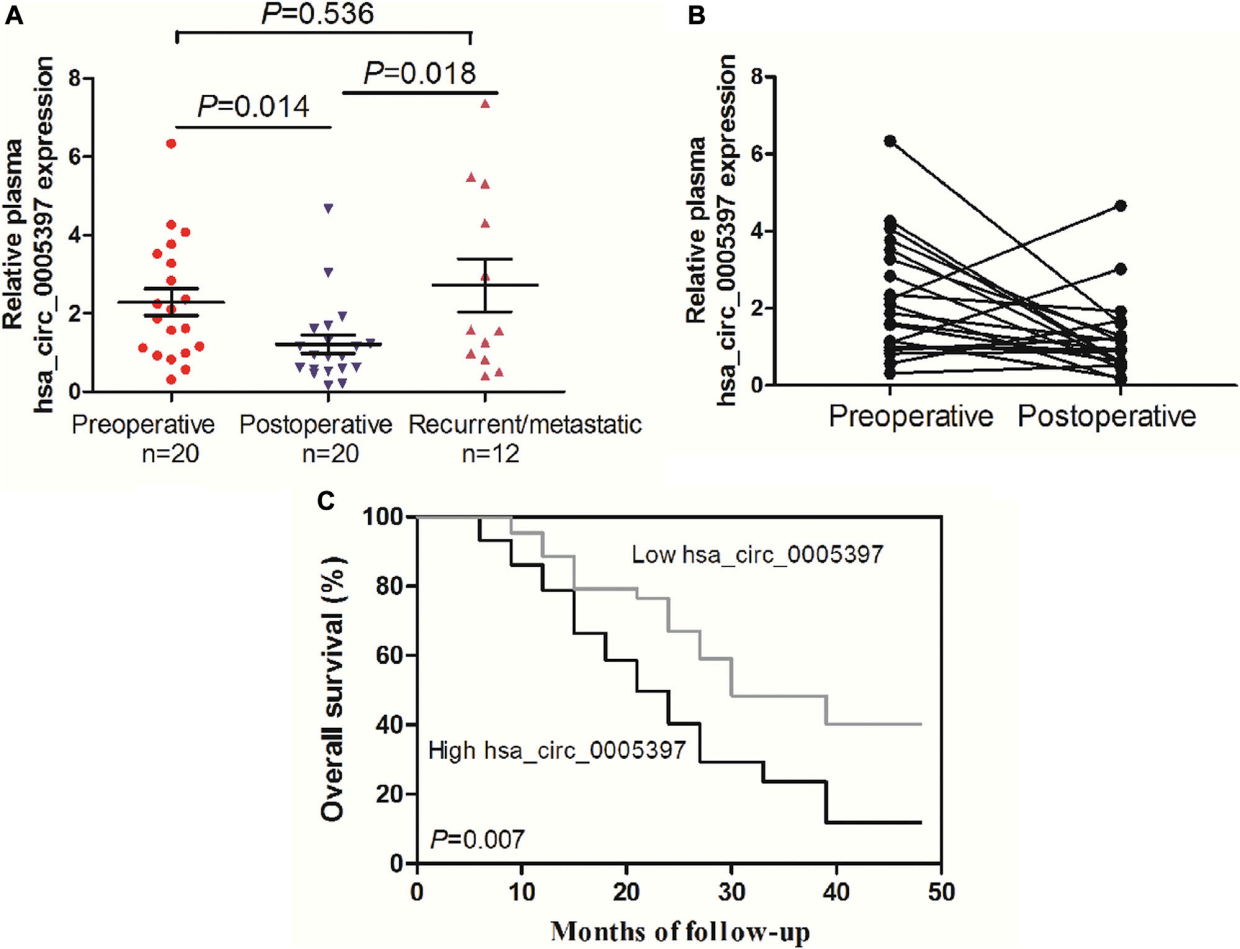
Dynamic analysis of plasma hsa_circ_0005397 and its prognosis value in patients with HCC. **(A, B)** The expression levels of plasma hsa_circ_0005397 were monitored from preoperative, postoperative and recurrent or metastatic HCC patients. **(C)** Kaplan–Meier survival curve analysis based on the plasma hsa_circ_0005397 expression levels of HCC patients.

We further performed dynamic analyses of plasma hsa_cic_0005397 in 20 HCC cases who had undergone surgical resection. As compared with preoperative group, plasma hsa_cic_0005397 level was drastically dropped in HCC patients after operation, *p* = 0.014 (Figures 6A,B). In total of 20 HCC cases, 12 cases had suffered from recurrence or metastasis. Notably, plasma hsa_cic_0005397 level was prominently elevated in recurrent or metastatic HCC cases, as compared with postoperative group, *p* = 0.018 (Figure 6A).

### Correlation of Plasma hsa_circ_0005397 With Prognosis of HCC Patients

To explore the prognostic value of plasma hsa_cic_0005397 in HCC patients, survival analysis was performed according to the follow-up data. The patients were categorized into low and high groups based on the median level = 1.87 of plasma hsa_cic_0005397. As shown in Figure 6C, overall survival in patients with high level of plasma hsa_cic_0005397 was shorter than those of low level group, *p* = 0.007.

## Discussion

HCC is a common cancer with high mortality. Percutaneous liver biopsy is usually used to confirm diagnosis of HCC, which is an invasive method (Tischfield et al., 2019). However, noninvasive testing is more acceptable in clinical practice. Serum AFP has been commonly used in clinical diagnosis of HCC over the past few decades, but its sensitivity and specificity are relatively unsatisfactory. To make up for the shortcomings of serum AFP, discovery of noninvasive, highly accurate and easily operable biomarkers is really needed for early detection of HCC and improvement of the prognosis of this deadly disease (Luo et al., 2019). Overwhelming evidence has demonstrated that circRNA is differentially expressed in HCC tissues (Bai et al., 2019; Hu et al., 2020). Some circRNAs are upregulated in HCC, such as circRHOT1, hsa_circ_0056836 and Circ-CDYL, etc. (Wang et al., 2019; Li et al., 2020; Wei et al., 2020), acting as oncogenic roles. Nevertheless, other circRNAs are downregulated in HCC, such as hsa_circ_0001649, circRNA-5692 and circTRIM33-12, etc. (Liu et al., 2019; Su et al., 2019; Zhang et al., 2019), acting as tumor suppressors. These circRNAs showed close correlations with carcinogenesis, progression and poor prognosis of HCC, indicating great potential as tissue-specific biomarkers for HCC. In addition, circRNA also stably exists in peripheral blood due to its circular structure which is not easily degraded by RNA exonuclease (Vilades et al., 2020). For example, Zhu et al. (2020) reported that plasma hsa_circ_0027089 could act as a diagnostic biomarker for hepatitis B virus-related HCC. Moreover, Yu et al. (2020) demonstrated that plasma hsa_circ_0000976, hsa_circ_0007750 and hsa_circ_ 0139897 could serve as a panel to diagnose hepatitis B virus-related HCC. These plasma circRNAs might have great clinical value in early detection, curative observation, and prognostic evaluation for HCC.

In the present study, we firstly found plasma hsa_circ_0005397 was over-expressed in patients with HCC than that in patients with benign liver lesions and healthy controls. Moreover, RNase R digestion assay, Sanger sequencing, and reverse transcription plasma RNA with random 6-mers or oligo (dT) primers were performed to identify the plasma hsa_circ_0005397. The results showed that hsa_circ_0005397 had a complete closed loop structure and was stable in the plasma of HCC patients. In addition, we further analyzed the origin of circulating hsa_circ_0005397 and found that hsa_circ_0005397 expression was very often boosted in HCC tumor tissues, HCC cell lines and increased in cell culture medium in a time-dependent manner, indicating that plasma hsa_circ_0005397 might be released from tumor cells into the blood circulatory system.

Next, ROC curve analysis showed that plasma hsa_cic_0005397 yielded an AUC of 0.737 (95% CI, 0.671–0.795), with the sensitivity of 82.0% and the specificity of 58.8%. As we all know, serum AFP and AFP-L3 have been widely used in clinical practice for screening and auxiliary diagnosing of HCC over the past few decades (Gao and Song, 2017; Kim et al., 2018; Yang et al., 2019). Although the specificity of plasma hsa_circ_0005397 was not as good as serum AFP and AFP-L3, its sensitivity was higher than serum AFP and AFP-L3, exhibiting that plasma hsa_cic_0005397 might be new potential diagnostic biomarker for HCC. Besides, the capability for differential diagnosis of benign and malignant liver diseases was further analyzed by the combination assay of plasma hsa_cic_0005397, serum AFP and AFP-L3. Our results disclosed that the combination of all the three biomarkers might improve the value of diagnosis and differential diagnosis for HCC.

Additionally, we also surveyed whether plasma hsa_circ_0005397 was associated with clinicopathological characteristics of HCC. The results displayed that high level of plasma hsa_circ_0005397 was closely associated with tumor size and TNM stage of HCC patients, suggesting that plasma hsa_circ_0005397 could help us predict the degree of malignancy and judge the progression of HCC. Except this, accumulating evidence has revealed that dynamic detection of circulating free nucleic acid could evaluate the postoperative status, including remission, recurrence or metastasis in patients with malignancy (Hao et al., 2014; Fan et al., 2018; Zong et al., 2019). Our dynamic monitoring uncovered that plasma hsa_circ_0005397 level of HCC patients was decreased after surgical resection, yet, was apparently increased in recurrent or metastatic HCC patients, indicating that plasma hsa_circ_0005397 could help to estimate the curative effect and predict recurrence or metastasis of HCC patients. Furthermore, we also corroborated that higher level of plasma hsa_cic_0005397 was positively correlated with shorter overall survival of HCC patients, showing its potential prognostic value for HCC.

Taken together, our current study, for the first time, has described in detail the clinical value of plasma hsa_cic_0005397 for HCC. We demonstrated that overexpressed plasma hsa_cic_0005397 might serve as a novel noninvasive biomarker to improve clinical diagnosis, as well as help us predict progression and assess prognosis of HCC. However, this was a preliminary estimation about the clinical utility of plasma hsa_cic_0005397 in HCC. Several limitations should be taken more attention, such as 1) all cases enrolled in this study only from a single institution; 2) relatively small sample size; 3) lack of long-term follow-up; 4) standardized detection of the circRNA by qRT-PCR. Consequently, in order to comprehensively explore potential value of plasma hsa_cic_0005397 in HCC clinical practice, further studies should be concentrated on multi-centered collaboration, a larger cohort of cases collection, long-term follow-up and methodological evaluation of circRNA detection. To sum up, plasma hsa_cic_0005397 warrants a promising candidate for early detection or screening AFP-negative HCC, monitoring treatment and prognosis of this deadly disease.

## Data Availability

The raw data supporting the conclusion of this manscript will be made available by the authors, without undue reservation.

## References

[B1] Bai N. Peng E. Xia F. Wang D. Li X. Li X. (2019). CircABCC2 regulates hepatocellular cancer progression by decoying MiR-665. J. Cancer 10, 3893–3898. 10.7150/jca.31362 31417632 PMC6692622

[B2] Ding Y. Liu K. Xu Y. Zhao Q. Lou S. Xiang X. (2020). Combination of inflammatory score/liver function and AFP improves the diagnostic accuracy of HBV-related hepatocellular carcinoma. Cancer Med. 9, 3057–3069. 10.1002/cam4.2968 32150664 PMC7196063

[B3] Ebbesen K. K. Hansen T. B. Kjems J. (2017). Insights into circular RNA biology. RNA Biol. 14, 1035–1045. 10.1080/15476286.2016.1271524 27982727 PMC5680708

[B4] Fan K. Ritter C. Nghiem P. Blom A. Verhaegen M. E. Dlugosz A. (2018). Circulating cell-free miR-375 as surrogate marker of tumor burden in merkel cell carcinoma. Clin. Cancer Res. 24, 5873–5882. 10.1158/1078-0432.CCR-18-1184 30061360 PMC6352975

[B5] Fu Y. Liu S. Zeng S. Shen H. (2019). From bench to bed: the tumor immune microenvironment and current immunotherapeutic strategies for hepatocellular carcinoma. J. Exp. Clin. Cancer Res. 38, 396. 10.1186/s13046-019-1396-4 31500650 PMC6734524

[B6] Gao J. Song P. (2017). Combination of triple biomarkers AFP, AFP-L3, and PIVAKII for early detection of hepatocellular carcinoma in China: Expectation. Drug Discov. Ther. 11, 168–169. 10.5582/ddt.2017.01036 28757516

[B7] Han L. Zhang X. Wang A. Ji Y. Cao X. Qin Q. (2020). A dual-circular RNA signature as a non-invasive diagnostic biomarker for gastric cancer. Front. Oncol. 10, 184. 10.3389/fonc.2020.00184 32154178 PMC7047344

[B8] Hao T. B. Shi W. Shen X. J. Qi J. Wu X. H. Wu Y. (2014). Circulating cell-free DNA in serum as a biomarker for diagnosis and prognostic prediction of colorectal cancer. Br. J. Cancer 111, 1482–1489. 10.1038/bjc.2014.470 25157833 PMC4200099

[B9] Hu Z. Q. Zhou S. L. Li J. Zhou Z. J. Wang P. C. Xin H. Y. (2020). Circular RNA sequencing identifies CircASAP1 as a Key regulator in hepatocellular carcinoma metastasis. Hepatology 72, 906–922. 10.1002/hep.3106810.1002/hep.31068 31838741

[B10] Huang X. He M. Huang S. Lin R. Zhan M. Yang D. (2019). Circular RNA circERBB2 promotes gallbladder cancer progression by regulating PA2G4-dependent rDNA transcription. Mol. Cancer 18, 166. 10.1186/s12943-019-1098-8 31752867 PMC6868820

[B11] Jie M. Wu Y. Gao M. Li X. Liu C. Ouyang Q. (2020). CircMRPS35 suppresses gastric cancer progression via recruiting KAT7 to govern histone modification. Mol. Cancer 19, 56. 10.1186/s12943-020-01160-2 32164722 PMC7066857

[B12] Kim H. Sohn A. Yeo I. Yu S. J. Yoon J. H. Kim Y. (2018). Clinical assay for AFP-L3 by using multiple reaction monitoring-mass spectrometry for diagnosing hepatocellular carcinoma. Clin. Chem. 64, 1230–1238. 10.1373/clinchem.2018.289702 29875214

[B13] Li Z. Liu Y. Yan J. Zeng Q. Hu Y. Wang H. (2020). Circular RNA hsa_circ_0056836 functions an oncogenic gene in hepatocellular carcinoma through modulating miR-766-3p/FOSL2 axis. Aging 12, 2485–2497. 10.18632/aging.102756 32048611 PMC7041754

[B14] Lin J. Cai D. Li W. Yu T. Mao H. Jiang S. (2019). Plasma circular RNA panel acts as a novel diagnostic biomarker for colorectal cancer. Clin. Biochem. 74, 60–68. 10.1016/j.clinbiochem.2019.10.012 31669510

[B15] Liu Z. Yu Y. Huang Z. Kong Y. Hu X. Xiao W. (2019). CircRNA-5692 inhibits the progression of hepatocellular carcinoma by sponging miR-328-5p to enhance DAB2IP expression. Cell Death Dis. 10, 900. 10.1038/s41419-019-2089-9 31776329 PMC6881381

[B16] Luo C. L. Rong Y. Chen H. Zhang W. W. Wu L. Wei D. (2019). A logistic regression model for noninvasive prediction of AFP-negative hepatocellular carcinoma. Technol. Cancer Res. Treat. 18, 153. 10.1177/1533033819846632 PMC6535757 31106685

[B17] Mak L. Y. Cruz-Ramón V. Chinchilla-López P. Torres H. A. LoConte N. K. Rice J. P. (2018). Global epidemiology, prevention, and management of hepatocellular carcinoma. Am. Soc. Clin. Oncol. Educ. Book 38, 262–279. 10.1200/EDBK_200939 30231359

[B18] Su Y. Xu C. Liu Y. Hu Y. Wu H. (2019). Circular RNA hsa_circ_0001649 inhibits hepatocellular carcinoma progression via multiple miRNAs sponge. Aging 11, 3362–3375. 10.18632/aging.101988 31137016 PMC6813922

[B19] Sun J. Zhao Y. Qin L. Li K. Zhao Y. Sun H. (2019). Metabolomic profiles for HBV related hepatocellular carcinoma including alpha-fetoproteins positive and negative subtypes. Front. Oncol. 9, 1069. 10.3389/fonc.2019.01069 31681602 PMC6803550

[B20] Tischfield D. J. Ackerman D. Noji M. Chen J. X. Johnson O. Perkons N. R. (2019). Establishment of hepatocellular carcinoma patient-derived xenografts from image-guided percutaneous biopsies. Sci. Rep. 9, 10546. 10.1038/s41598-019-47104-9 31332214 PMC6646301

[B21] Vilades D. Martínez-Camblor P. Ferrero-Gregori A. Bär C. Lu D. Xiao K. (2020). Plasma circular RNA hsa_circ_0001445 and coronary artery disease: performance as a biomarker. FASEB J 34, 4403–4414. 10.1096/fj.201902507R 31999007

[B22] Wang L. Long H. Zheng Q. Bo X. Xiao X. Li B. (2019). Circular RNA circRHOT1 promotes hepatocellular carcinoma progression by initiation of NR2F6 expression. Mol. Cancer 18, 119. 10.1186/s12943-019-1046-7 31324186 PMC6639939

[B23] Wei Y. Chen X. Liang C. Ling Y. Yang X. Ye X. (2020). A noncoding regulatory RNAs network driven by circ-CDYL acts specifically in the early stages hepatocellular carcinoma. Hepatology 71, 130–147. 10.1002/hep.30795 31148183

[B24] Xiao Y. Liu G. Sun Y. Gao Y. Ouyang X. Chang C. (2020). Targeting the estrogen receptor alpha (ERα)-mediated circ-SMG1.72/miR-141-3p/Gelsolin signaling to better suppress the HCC cell invasion. Oncogene 39, 2493–2508. 10.1038/s41388-019-1150-6 31996784

[B25] Yang T. Xing H. Wang G. Wang N. Liu M. Yan C. (2019). A novel online calculator based on serum biomarkers to detect hepatocellular carcinoma among patients with hepatitis B. Clin. Chem. 65, 1543–1553. 10.1373/clinchem.2019.308965 31672853

[B26] Yao Z. Xu R. Yuan L. Xu M. Zhuang H. Li Y. (2019). Circ_0001955 facilitates hepatocellular carcinoma (HCC) tumorigenesis by sponging miR-516a-5p to release TRAF6 and MAPK11. Cell Death Dis. 10, 945. 10.1038/s41419-019-2176-y 31822654 PMC6904727

[B27] Yin Y. Long J. He Q. Li Y. Liao Y. He P. (2019). Emerging roles of circRNA in formation and progression of cancer. J. Cancer 10, 5015–5021. 10.7150/jca.30828 31602252 PMC6775606

[B28] Yu J. Ding W. B. Wang M. C. Guo X. G. Xu J. Xu Q. G. (2020). Plasma circular RNA panel to diagnose hepatitis B virus-related hepatocellular carcinoma: a large-scale, multicenter study. Int. J. Cancer 146, 1754–1763. 10.1002/ijc.32647 31456215

[B29] Zhang P. F. Wei C. Y. Huang X. Y. Peng R. Yang X. Lu J. C. (2019). Circular RNA circTRIM33-12 acts as the sponge of MicroRNA-191 to suppress hepatocellular carcinoma progression. Mol. Cancer 18, 105. 10.1186/s12943-019-1031-1 31153371 PMC6545035

[B30] Zhu K. Zhan H. Peng Y. Yang L. Gao Q. Jia H. (2020). Plasma hsa_circ_0027089 is a diagnostic biomarker for hepatitis B virus-related hepatocellular carcinoma. Carcinogenesis 41, 296–302. 10.1093/carcin/bgz154 31535687 PMC7221502

[B31] Zhu Y. J. Zheng B. Luo G. J. Ma X. K. Lu X. Y. Lin X. M. (2019). Circular RNAs negatively regulate cancer stem cells by physically binding FMRP against CCAR1 complex in hepatocellular carcinoma. Theranostics 9, 3526–3540. 10.7150/thno.32796 31281495 PMC6587157

[B32] Zong W. Feng W. Jiang Y. Ju S. Cui M. Jing R. (2019). Evaluating the diagnostic and prognostic value of serum long non-coding RNA CTC-497E21.4 in gastric cancer. Clin. Chem. Lab. Med. 57, 1063–1072. 10.1515/cclm-2018-0929 30763257

